# Alpha Waves as a Neuromarker of Autism Spectrum Disorder: The Challenge of Reproducibility and Heterogeneity

**DOI:** 10.3389/fnins.2018.00662

**Published:** 2018-10-01

**Authors:** Aline Lefebvre, Richard Delorme, Catherine Delanoë, Frederique Amsellem, Anita Beggiato, David Germanaud, Thomas Bourgeron, Roberto Toro, Guillaume Dumas

**Affiliations:** ^1^Department of Child and Adolescent Psychiatry, Assistance Publique-Hôpitaux de Paris, Robert Debré Hospital, Paris, France; ^2^Human Genetics and Cognitive Functions Unit, Institut Pasteur, Paris, France; ^3^CNRS UMR 3571 Genes, Synapses and Cognition, Institut Pasteur, Paris, France; ^4^Sorbonne Paris Cité, Human Genetics and Cognitive Functions, University Paris Diderot, Paris, France; ^5^Neurophysiology Department, Assistance Publique-Hôpitaux de Paris, Robert Debré Hospital, Paris, France; ^6^Pediatric Neurology Department, Assistance Publique-Hôpitaux de Paris, Robert Debré Hospital, Paris, France

**Keywords:** child psychiatry, reproducibility, biomarker, spectral analysis, autism spectrum disorders

## Abstract

**Background:** There is no consensus in the literature concerning the presence of abnormal alpha wave profiles in patients with autism spectrum disorder (ASD). This may be due to phenotypic heterogeneity among patients as well as the limited sample sizes utilized. Here we present our results of alpha wave profile analysis based on a sample larger than most of those in the field, performed using a robust processing pipeline.

**Methods:** We compared the alpha waves profiles at rest in children with ASD to those of age-, sex-, and IQ-matched control individuals. We used linear regression and non-parametric normative models using age as covariate forparsing the clinical heterogeneity. We explored the correlation between EEG profiles and the patient’s brain volumes, obtained from structural MRI. We automatized the detection of the alpha peak and visually quality controled our MRI measurements. We assessed the robustness of our results by running the EEG preprocessing with two different versions of Matlab as well as Python.

**Results:** A simple linear regression between peak power or frequency of the alpha waves and the status or age of the participants did not allow to identify any statistically significant relationship. The non-parametric normative model (which took account the non-linear effect of age on the alpha profiles) suggested that participants with ASD displayed more variability than control participants for both frequency and amplitude of the alpha peak (*p* < 0.05). Independent of the status of the individual, we also observed weak associations (uncorrected *p* < 0.05) between the alpha frequency, and the volumes of several cortical and subcortical structures (in particular the striatum), but which did not survive correction for multiple testing and changed between analysis pelines.

**Discussions:** Our study did not find evidence for abnormal alpha wave profiles in ASD. We propose, however, an analysis pipeline to perform standardized and automatized EEG analyses on large cohorts. These should help the community to address the challenge of clinical heterogeneity of ASD and to tackle the problems of reproducibility.

## Introduction

Autism spectrum disorders (ASD), which affect 1–2% of the general population, are characterized by impairments in social communication associated with repetitive, stereotyped, or ritualistic behaviors (American Psychiatric Association, 2000). Despite the unifying definition, ASD is a highly heterogeneous condition since patients with ASD display a variable clinical presentation ranging from mild to severe impairments and are frequently associated with comorbid disorders including intellectual developmental disability, developmental language or coordination disorders, or attention deficit and hyperactivity disorder (ADHD) (Gillberg, 2010). Besides, genetic and environmental causes and risk factors appear highly heterogeneous in ASD too. Considering the clinical heterogeneity and the prevalence of ASD, the identification of biomarkers, especially neuromarkers, is a public health issue.

Neuroimaging raised new hope in our ability to further understand the biological mechanisms associated with ASD. Functional MRI (fMRI), for instance, allowed to study brain activity during rest and various tasks (Raichle et al., 2001; Di Martino et al., 2013; Hahamy et al., 2015; Crippa et al., 2016). In ASD, results from the last 10 years depicted a mixed patterns of dysconnectivity with sometimes an under-connectivity, especially in networks involving frontal regions, and sometimes over-connectivity, for instance in the bilateral temporo-limbic regions (Castelli et al., 2002; Just et al., 2004; Courchesne and Pierce, 2005; Weng et al., 2010; Di Martino et al., 2013). Finally large scales studies, based on data-sharing initiatives, such as the ABIDE network, reported both under- and over-connectivity in ASD (Jung et al., 2017). Recent results also support a higher inter-subject heterogeneity in the spatial patterns of functional connectivity across ASD individuals, probably accounting the variation of results in previous studies (Hahamy et al., 2015).

Electroencephalography (EEG) has been also used to explore the neural correlates of brain functions, for even longer. Multiple studies have uncovered EEG patterns that could relate to social communication abilities. For example, the alpha rhythm (8–12 Hz), the most dominant rhythm during wakefulness, has been associated with precise timing of visual perception (Klimesch et al., 1998; Palva and Palva, 2011), consciousness state (Engemann and Gramfort, 2015), sensory cognitive inhibition (Pfurtscheller et al., 1996, 2006), and even to social coordination (Tognoli et al., 2007; Dumas et al., 2010; Kelso et al., 2013). At the physiological level, alpha oscillations may modulate the transfer of information in the thalamo-cortical and cortico-cortical networks but also facilitate and gate the external sensory perception (Klimesch et al., 2007; Busch et al., 2009; Jensen and Mazaheri, 2010; Amzica and Lopes da Silva, 2012). In the 70s, studies in typically developing (TD) participants reported developmental trajectory of alpha waves with the frequency increasing and the power decreasing until around 10 years old, but those results were not questioned nor replicated since (Matousek and Petersén, 1971; Petersén and Eeg-Olofsson, 1971). In ASD, early reports also described less alpha in low-functioning autistic children (Cantor et al., 1986). More recently, some authors reported that the alpha power was positively correlated at rest with the intensity of social deficit (measured with the Social Responsiveness Scale) (Cornew et al., 2012; Edgar et al., 2015) although several studies showed inconsistent results (Keehn et al., 2017). For example, Oberman et al. (2005) reported in participant with ASD, a normal alpha amplitude at rest in both the parietal cortex and the superior temporal sulcus; both being involved in action perception and understanding and considered as part of the mirror neuron system (Rizzolatti et al., 2001). Notice that since the 10 Hz oscillations over the primary somatosensory cortex have been traditionally called Rolandic or mu rhythm, authors now tend to use alpha-mu as a general term, especially if the task contains action execution or observation. Indeed, during action execution or observation, alpha-mu is suppressed (Gastaut, 1952; Hobson and Bishop, 2017). In ASD, the lack of similar effect during action observation lead to the hypothesis that autism was linked to a dysfunction of the MNS – the so-called “broken mirror hypothesis” (Oberman et al., 2005) – and that this dysfunction could be a biomarker of ASD (Dickinson et al., 2017; Keehn et al., 2017).

Alpha-mu abnormalities in ASD (Cantor et al., 1986; Dawson et al., 1995; Chan and Leung, 2006; Murias et al., 2007; Machado et al., 2015; Shephard et al., 2018) may be explained by multiple factors: (1) at the intrinsic level, the major phenotypic heterogeneity in patients with ASD with a combination of comorbidities (for review, see Jeste et al., 2015), etiological heterogeneity, and risk factors, but also variance factors such as the age of the participants or cerebral volume; and (2) at the extrinsic level, the variations of methods across studies (Hobson and Bishop, 2016). For instance, studies tend to use different frequency bands and linear approaches to extract alpha-mu characteristics. Those methods (e.g., fast Fourier transform or FFT) require stationary signals (Wang et al., 2013) while EEG dynamics is nonlinear and complex. Pipelines of analysis may thus rely on more advanced signal processing approaches (Bigdely-Shamlo et al., 2015). However, the choice frequency band may be the most important source of bias in the results reported in ASD (Dumas et al., 2014b). Indeed, the initial reports of abnormal alpha-mu suppression in ASD may result from an analysis considering alpha waves as a homogeneous phenomenon covering the 8–12/13 Hz frequency band, although it can be functionally segregated in two distinct sub-bands, 8–10 and 10–12/13 Hz (Bazanova and Vernon, 2014). When considering only the upper sub-band, a suppression of the alpha waves in the sensorimotor cortex during response to motor observation was observed in both TD and ASD participants (Dumas et al., 2014b). Finally, the discrepancies in the literature may also result from spatial effects. Indeed, source reconstructions revealed an abnormal alpha-mu pattern in ASD, with simultaneously a decrease in occipito-parietal regions and an increase in frontal regions, resulting in an apparent absence of mu-suppression over the central regions at the scalp level (Dumas et al., 2014b).

To quantify the impact of heterogeneity both ASD participants and methodological choices, we developed a systematic pipeline of analysis across different versions of software. Concerning clinical heterogeneity, specifically the impact of developmental variation, we used normative models using age as a clinical covariate (Marquand et al., 2016; Bethlehem et al., 2018). Concerning variability of methodological choices, we control the reproducibility of manual alpha peak detection between human observers and automatic alpha peak detection between software. We especially compared analysis pipelines across two versions of Matlab and Python. We finally tested if the variability of the alpha peak characteristics could be explained by a similar variability in structural brain volumes (cortical and subcortical structures). We specifically explored the correlation with thalamic volume, since thalamus has been proposed as a core pacemaker for the alpha waves (Edgar et al., 2015).

## Materials and Methods

### Participants

A sample of 88 individuals composed of 44 participants with ASD and their sex-, age-, and IQ-matched TD participants (*N* = 44) were enrolled in the study (**Table 1** and **Supplementary Figure S1**). All participants were from the Paris Autism Research International Sibpair (PARIS) consortium cohort and recruited at the Child and Adolescent Psychiatry Department, Robert Debré Hospital, Paris (France). Patients with ASD were included after a systematic clinical and medical check-up including negative blood tests results for Fragile-X and exclusion of participants carrying a large deletion over 2 Mb detected by the Illumina 700K SNPs array, in an attempt to further analysis combining genetic, MRI, and EEG data. Final diagnosis of ASD was based on DSM-IV TR criteria and made by summing the information from the Autism Diagnosis Interview – Revised (ADI-R) (Lord et al., 1994), the Autism Diagnostic Observation Scale (ADOS) (Lord et al., 2000), and data from clinical reports from expert in the field. In multiplex families, only index cases were included in the present study to ensure unbiased estimate of the alpha waves’ quantification. Psychiatric comorbidities according to DSM-IV-TR were screened with a semi-structured direct interview, the Schedule for Affective Disorders, and Schizophrenia for School-Age Children, Present and Lifetime Version (K-SADS-PL). Intellectual functioning of all participants was estimated with the Raven’s Progressive Matrices or with the Wechsler Intelligence Scales.

**Table 1:** Clinical and demographic characteristics of probands with ASD and their controls enrolled in the study for alpha waves analysis in patients and TD participants.

	ASD (*n* = 44)	TD (*n* = 44)
Males, % (no.)	75% (33)	75% (33)
Current age, months *m* (*SD*)	116.01 (43.6)	116.05 (45.8)
Non-verbal IQ (*SD*)	91.2 (24.0)	93.7 (23.6)
ADI-R subdomain scores		
Social	16.4 (9.4)	–
Communication	12.4 (7.8)	–
Repetitive behavior	5.5 (3.6)	–
ADOS – two subdomain scores		
Communication	4.8 (1.9)	–
Social	3.5 (3.0)	–
Repetitive behaviors	1.6 (1.5)	–
SRS total score (*t*-score)	74.8 (11.8)	–

*ASD, autism spectrum disorder; TD, typically developing participants; ADI-R, autism diagnostic interview – revisited; ADOS, autism diagnostic observation schedule.*

Participants from the control group were from the general population. All participants with a personal or a familial history of ASD were not included. Participants were TD children. They never reported any speech therapy, psychiatric, or neurological follow-up or a personal history of traumatic brain injury, severe prematurity (<1850 g at birth), or epilepsy.

### Ethics Statement

This study was carried out in accordance with the recommendations of the local ethics committee of Hospital Robert Debré. All participants gave written informed consent in accordance with the Declaration of Helsinki. The protocol was approved by the Inserm Ethics Committee (study approval No. 08-029).

### Electroencephalogram Acquisition and Data Extraction

Digital 10-channel EEG (FP1/2, F7/8, T5/6, O1/2, C3/4) was recorded using a Nihon-Kohden (Inc., Tokyo, Japan) system with the electrodes positioned according to the International 10–20 system (Sharbrough et al., 1994). Unipolar leads tracings were taken, regarding both sides of the ear as the reference electrode (Sharbrough et al., 1994). The EEG activity was acquired using a linked ears reference, sampled at 500 Hz, and filtered offline between 1 and 120 Hz. Impedance was kept below 5 kΩ. Vigilance-controlled recordings were made according to usual clinical standards, including a 10-min resting eyes-closed state, 1 min of alternate 10 s-eyes open/eyes closed conditions, 3 min of hyperventilation, 10 min of recovery (post-hyperventilation), and 4-min photic stimulation (from 0.5 to 60 Hz). After recording, whole raw data in Nihon Koden format were converted into an EEG EDF+ format allowing their analysis into Matlab^TM^. Two distinct toolboxes BIOSIG (Vidaurre et al., 2011) and EEGlab (Delorme and Makeig, 2004), and a custom script for reading EDF+ files (Duann and Hatz, 2012) were used to perform the pre-processing and processing of the data (**Supplementary Figure S2**). The final processing pipeline used the EEGlab 13.4.4b version with Matlab 2014b.

The power line interferences were removed with a Finite Impulse Response (FIR) comb filter and electrodes with signal-to-noise ratio over three standard errors were interpolated by spherical splines. Then, FFTs on 1 s sliding windows gave spectra for all electrodes. Detection of alpha peak was automatized by computing the spectra differences between eyes closed and eyes open periods, and then, by selecting among the occipital and parietal sensors the one exhibiting the maximum peak. However, to ensure the quality of the alpha peak detection, we performed a visual inspection of each EEG spectrum by two independent raters. Each spectrum was rated on a 4-point Likert scale from 1 (good quality) to 4 (unusable data). For the final analysis, we excluded the data for which one rater (or both) considered the spectrum as unusable (score = 4) (**Supplementary Figure S1**). The inter-rater agreement for qualitative items was computed with the Bangdiwala’s test using R version 3.3.3 (2017-03-06).

### Non-parametric Normative Modeling

The normative modeling (NM) approach has been introduced in psychiatry as an alternative to the traditional case–control contrasts. The idea consists of fitting a mathematical distribution to a population of control group, considering its heterogeneity across multiple dimensions. Once this model is set, it is possible to assign to every participant, including the controls, a score measuring their distance from the normative model (Marquand et al., 2016). In a nutshell, NM provides a metric similar to a *Z*-score, but accounts for the underlying structure of the population across multiple covariates. The original version of NM uses Gaussian Processes (GPs) to model the distribution of control group measures. The major advantage is the ability to use Bayesian optimization in the fit of those GP. Other family of functions can be used but for data with strong heterogeneity or with non-classic distribution, the obtained results can become misleading. Here we used non-parametric version using LOESS Curve Fitting (Local Polynomial Regression). LOESS is a nonparametric method that uses local weighted regression to fit a smooth curve through points in a scatter plot. The procedure was originally proposed as Locally Weighted Scatter-plot Smoother (LOWESS) by Cleveland (1979) and further developed by Cleveland and Devlin (1988). The normative model was calculated by approximating the TD participants data with a polynomial function of age (smoothing kernel of 2 years). Python code is available at https://github.com/GHFC/SoNeTAA/.

### Intracranial and Brain Volume Estimations Based on Magnetic Resonance Imaging

For participants with ASD included in this study, MRI data were collected using the following parameters: spoiled gradient recalled echo (SPGR), 1 mm isotropic, repetition time (TR) = 25 ms, echo time (TE) = 6 ms, flip angle = 30°. To estimate the intracranial volume, the different datasets were first reoriented to correspond with the orientation of the MNI152 atlas. The brain was removed from the skull using AFNI tools (Cox, 1996), and linearly normalized to the (skull-less) MNI152 atlas using FSL tools (Jenkinson et al., 2002; Smith et al., 2004). We then used the affine matrix of this transformation to initialize the linear normalization of the reoriented datasets (with skull) to the MNI152 atlas (with skull). We used the inverse of the determinant of the affine matrix produced by this transformation as an estimation of the intracranial volume, as in Buckner et al. (2004). All steps of the process were visually inspected for accuracy using in-house software. In the cases where the skull stripping was inappropriate (the most frequent type of failure of the automatic segmentation procedure), we manually corrected the brain extraction using in-house software, and relaunched the processing pipeline. The estimation of brain volume was obtained by segmenting automatically the gray and white matter. We then labeled the frontal, parietal, occipital, temporal lobes, and subcortical structures by non-linearly warping the individual datasets into an atlas, using FSL tools (Zhang et al., 2001; Smith et al., 2004) and our own software (Toro et al., 2009). Brain volume was calculated as the sum of gray and white matter in the frontal, parietal, occipital, and temporal lobes excluding ventricles and subcortical structures and the intracranial volume estimated as the inverse of the determinant of the linear transformation matrix of each participant’s brain into the MNI template excluding the skull. We used a tool to visually control the accuracy of the segmentations: https://github.com/neuroanatomy/QCApp-vsub. The participants for whom the segmentation not fulfilled this visual quality control were excluded. After quality control and coupling with EEG data, neuroanatomic analysis was done for 20 patients with ASD (**Supplementary Figure S1**).

### Statistical Analysis

After quality control with Bangdiwala’s test (**Supplementary Figure S3**), we analyzed data for 26 participants with ASD (128.11 m.o. ± 37.6) and for 33 participants with typical development (125.5 m.o. ± 39.6) (**Table 2** and **Supplementary Figure S1**). We explored the statistical difference for alpha peak frequency and power between the two groups. An analysis of variance was used to control the effect on age, status (affected or not), and the interaction age ^∗^ status on the frequency and the power of alpha peak across groups. Normative models were computed with Python 2.7. We finally explored the relationship between the frequency and the power of the alpha peak with each brain neuroanatomic structures. Statistical analyses were performed with JMP Pro 11.2.0. SAS^TM^.

**Table 2:** Clinical and demographic characteristics of probands with ASD and their controls analyzed in the study for alpha waves analysis in patients and TD participant after control quality.

	ASD (*n* = 26)	TD (*n* = 33)
Males, % (no.)	100% (26)	100% (33)
Current age, months *m* (*SD*)	128.1 (37.6)	125.5 (39.6)

To check the robustness of our EEG results, we used not only two distinct versions of Matlab but also the Python language, since we observed divergence in the preliminary tests with Matlab. We first performed our analysis with Matlab 2014b with the signal processing toolbox and the 13.4.4b version of the EEGlab toolbox. We then compared our results to those obtained with Matlab 2013a without the signal processing toolbox, using the 12.0.2.6b version of the EEGlab toolbox, and to those obtained using Python. For this later analysis, we used Python 2.7 and the MNE Python library (Gramfort et al., 2013). Key steps of the pipeline were kept as identical as possible between Matlab and Python pipelines. Both versions, including the script for non-parametric normative model are available on our GitHub repository: https://github.com/GHFC/SoNeTAA/.

## Results

### Alpha Waves in Patients and Typically Developing Participants

**FIGURE 1:**
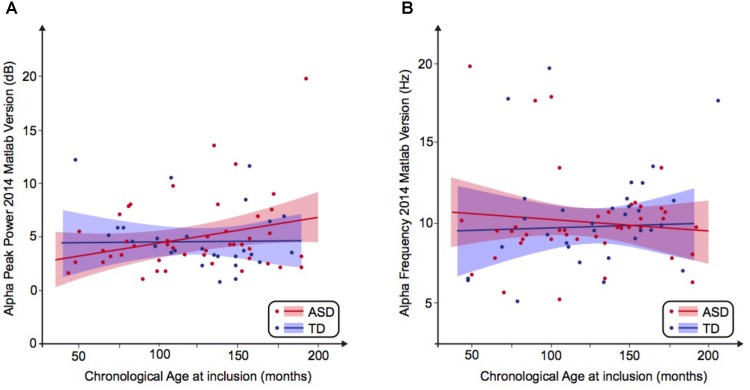
Alpha waves neurodevelopmental trajectory analyzed by Matlab 2014. **(A)** Alpha peak power (dB) depending on age (in months). **(B)** Alpha frequency (Hz) depending on age (in months). ASD, autism spectrum disorder; TD, typically developing participants.

The alpha peak characteristics extracted with Matlab 2014 and EEGlab 13.4.4b are summarized in **Table 3**. We did not observe any significant difference for alpha peak frequency and power between the groups. We were also unable to detect any significant variance differences between groups for both alpha frequency (*F* two-sided = 0.41; *p* = 0.66) and alpha peak power (*F* two-sided = 0.42; *p* = 0.66) (**Table 3**). An analysis of the variance (ANOVA) of the frequency of the alpha peak was performed including age, status, and interaction age ^∗^ status as independent variables (*F* = 0.23; *R*^2^ = 0.01; *p* = 0.87). There was no significant effect of the status (*F* = 0.16; *p* = 0.69), nor of the age (*F* = 0.07; *p* = 0.87) and the interaction age ^∗^ status on the alpha frequency (*F* = 0.16; *p* = 0.57) (**Figure 1**). A similar ANOVA analysis was also run for the power of the alpha peak (*F* = 1.21; *R*^2^ = 0.05; *p* = 0.31) and showed no significant effect of age at inclusion (*F* = 1.36; *p* = 0.24), nor of the status or of the interaction between age ^∗^ status (*F* = 0.22; *p* = 0.63; *F* = 1.18; *p* = 0.28, respectively).

**Table 3:** Alpha waves characteristics (Matlab 2014 – EEGlab13.4.4b).

		ASD	TD	*T*-ratio (*p*-value)
Alpha frequency (Hz)	Mean (*SD*)	9.8 (3.1)	10.6 (2.9)	0.4 (0.7)
Alpha peak power (dB)	Mean (*SD*)	4.5 (3.3)	4.9 (3.7)	0.42 (0.7)

*ASD, autism spectrum disorder; TD, typically developing participants.*

We then performed a simple linear regression to model the relationship between the frequency or the peak power of the alpha waves and the age of the participant (**Figures 1A,B**). We observed no significant effect of age on the alpha frequency in TD participants [alpha frequency = 10.99–0.01 ^∗^ age (*R*^2^ = 0.01; *F* = 0.49; *p* = 0.49)] nor in the ASD group [alpha frequency = 9.41 + 0.003 ^∗^ age (*R*^2^ = 0.001; *p* = 0.85)]. We similarly observed no significant effect of age on the alpha peak power in TD participants [alpha peak power = 1.95 + 0.02 ^∗^ age (*R*^2^ = 0.08; *p* = 0.08)] nor in the ASD group [alpha peak power = 4.43 + 0.001 ^∗^ age (*R*^2^ = 0.0001; *p* = 0.96)]. These results suggested the variability of the alpha peak power and frequency prevents any conclusion on the difference between patients with ASD and TD participants.

### Alpha Waves Normative Modeling in Patients and Typically Developing Participants

**FIGURE 2:**
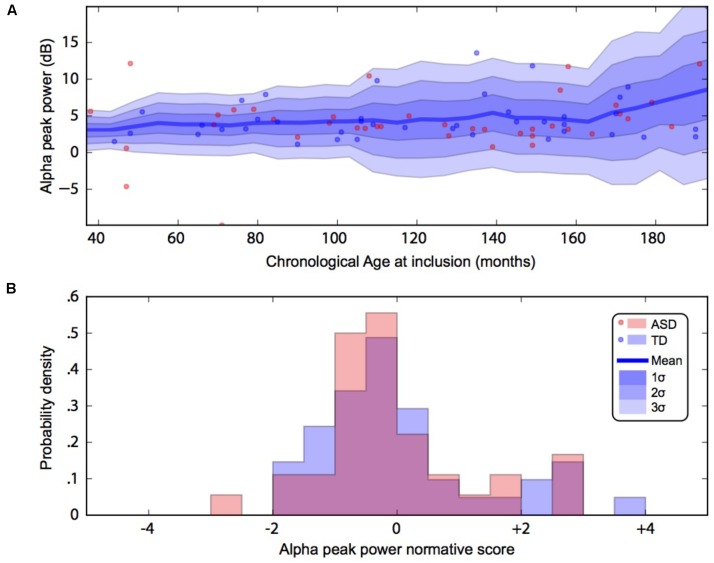
Alpha waves normative modeling in patients and TD participants: effect of age heterogeneity on alpha peak power. **(A)** Shows the raw data with the non-parametric normative model overlaid in blue. **(B)** Summarises the normative scores with an histogram. ASD, autism spectrum disorder; TD, typically developing participants.

**FIGURE 3:**
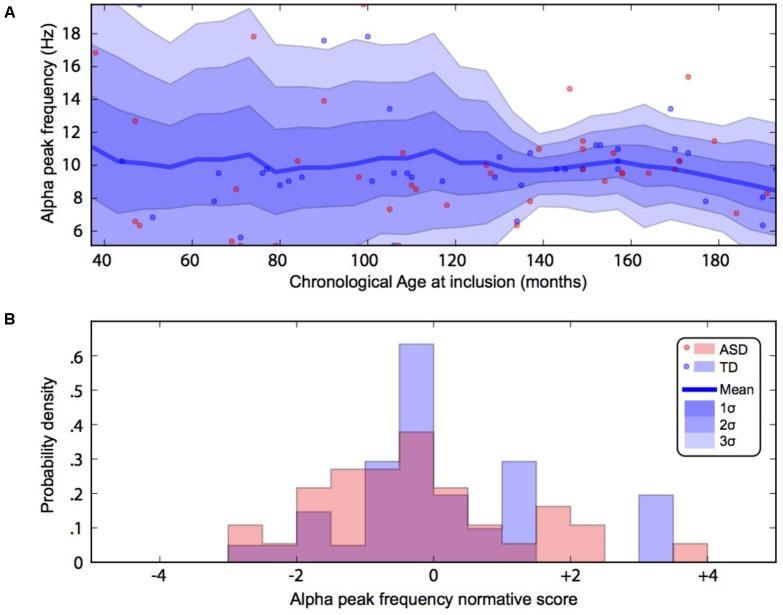
Alpha waves normative modeling in patients and TD participants: effect of age heterogeneity on alpha frequency. **(A)** Shows the raw data with the non-parametric normative model overlaid in blue. **(B)** Summarises the normative scores with an histogram. ASD, autism spectrum disorder; TD, typically developing participants.

By using a linear method approach, we did not replicate previous findings suggesting a developmental effect on alpha characteristics, i.e., an increase of the alpha frequency and power with the age of the participant. The potential non-linear effects of age on the alpha characteristics may account for the lack of association we reported with linear methods. We thus used a normative model to better consider the effect of age heterogeneity in our sample and its potential non-linear effect on alpha characteristics (**Figures 2**, **3**). All participants with alpha characteristics fit the normative model well, including the oldest participants whatever their status. We identified several individuals who were outliers within the distribution but no statistical deviation from expected proportions in alpha frequency (Fisher exact, *p* = 0.08) or amplitude (Fisher exact, *p* = 0.92). When quantifying the degree of deviation from the normal distribution, we observed that participants with ASD displayed more variability around the normal distribution than control participants for both frequency (*F* = 2.25, *p* < 0.05) and amplitude (*F* = 2.72, *p* < 0.05).

### Alpha Waves and Structural Brain Volume Correlations in ASD

We then compared the alpha characteristics (based on Matlab 2014b–EEGlab 13.4.4b) to the brain structure volumes. This analysis was performed only in the participant for which one had excellent to good EEG recording quality (i.e., participants with at least one unusable rating were excluded; *N* = 32). We performed a simple linear regression to model the relationship between the peak frequency or the power of alpha waves and each structural brain volumes (**Supplementary Figure S4**). There was no significant effect of each structural brain volumes on the alpha peak power, but we observed a significant effect on alpha frequency for: the left putamen volume [alpha frequency = 19.03 ^∗^ age (*R*^2^ = 0.13; *p* = 0.04)], the left accumbens nucleus volume [alpha frequency = 16.92-0.01 ^∗^ age (*R*^2^ = 0.17; *p* = 0.002)], the left parietal gray matter volume [alpha frequency = 27.3–0.0002 ^∗^ age (*R*^2^ = 0.15; *p* = 0.02)], the left occipital gray matter volume (alpha frequency = 20.19–0.0002 ^∗^ left occipital gray matter volume, *R*^2^ = 0.14, *p* = 0.03), the right occipital gray matter volume [alpha frequency = 21.04–0.0002-^∗^ age (*R*^2^ = 0.12; *p* = 0.05)], the left subcortical white matter [alpha frequency = −2.57-0.0002 ^∗^ age (*R*^2^ = 0.2; *p* = 0.009)], the right subcortical white matter volume [alpha frequency = −0.99-0.0002 ^∗^ age (*R*^2^ = 0.2; *p* = 0.008)] (see **Table 4** for a summary).

**Table 4:** Bivariate regression analysis regarding the association between alpha characteristics (frequency and power) analyzed with Matlab 2014, Matlab 2013, and Python related to neuroanatomic volumes.

		Alpha frequency			Alpha peak power		
		Matlab 2014	Matlab 2013	Python	Matlab 2014	Matlab 2013	Python
Intracranial brain volume		*R*^2^ = 0.01	*R*^2^ = 0.0001	*R*^2^ = 0.12	*R*^2^ = 0.03	*R*^2^ = 0.01	*R*^2^ = 0.11
Total brain volume		*R*^2^ = 0.08	*R*^2^ = 0.09	*R*^2^ = 0.15	*R*^2^ = 0.003	*R*^2^ = 0.04	*R*^2^ = 0.04
Thalamic volume	Left	*R*^2^ = 0.03	*R*^2^ = 0.05	*R*^2^ = 0.48	*R*^2^ = 0.002	*R*^2^ = 0.001	*R*^2^ = 0.05
	Right	*R*^2^ = 0.004	*R*^2^ = 0.05	*R*^2^ = 0.38	*R*^2^ = 0.0001	*R*^2^ = 0.001	*R*^2^ = 0.05
Caudate volume	Left	*R*^2^ = 0.02	*R*^2^ = 0.03	*R*^2^ = 0.35	*R*^2^ = 0.01	*R*^2^ = 0.06	*R*^2^ = 0.19
	Right	*R*^2^ = 0.006	*R*^2^ = 0.04	*R*^2^ = 0.27	*R*^2^ = 0.005	*R*^2^ = 0.08	*R*^2^ = 0.11
Putamen volume	Left	*R*^2^ = 0.13^∗^	*R*^2^ = 0.04	*R*^2^ = 0.11	*R*^2^ = 0.002	*R*^2^ = 0.09	*R*^2^ = 0.11
	Right	*R*^2^ = 0.08	*R*^2^ = 0.03	*R*^2^ = 0.17	*R*^2^ = 0.0003	*R*^2^ = 0.03	*R*^2^ = 0.0003
Pallidum volume	Left	*R*^2^ = 0.01	*R*^2^ = 0.007	*R*^2^ = 0.41	*R*^2^ = 0.0003	*R*^2^ = 0.0009	*R*^2^ = 0.0006
	Right	*R*^2^ = 0.002	*R*^2^ = 0.012	*R*^2^ = 0.28	*R*^2^ = 0.02	*R*^2^ = 0.007	*R*^2^ = 0.19
Hippocampic volume	Left	*R*^2^ = 0.03	*R*^2^ = 0.05	*R*^2^ = 0.28	*R*^2^ = 0.05	*R*^2^ = 0.0002	*R*^2^ = 0.13
	Right	*R*^2^ = 0.06	*R*^2^ = 0.05	*R*^2^ = 0.12	*R*^2^ = 0.01	*R*^2^ = 0.013	*R*^2^ = 0.02
Amygdala volume	Left	*R*^2^ = 0.0002	*R*^2^ = 0.02	*R*^2^ = 0.36	*R*^2^ = 0.002	*R*^2^ = 6.7 × 10^−5^	*R*^2^ = 0.09
	Right	*R*^2^ = 0.006	*R*^2^ = 0.02	*R*^2^ = 0.38	*R*^2^ = 0.005	*R*^2^ = 5.3 × 10^−5^	*R*^2^ = 0.05
Accumbens nucleus volume	Left	*R*^2^ = 0.17^∗^	*R*^2^ = 0.07	*R*^2^ = 0.18	*R*^2^ = 0.005	*R*^2^ = 0.06	*R*^2^ = 0.09
	Right	*R*^2^ = 0.07	*R*^2^ = 0.01	*R*^2^ = 0.24	*R*^2^ = 0.002	*R*^2^ = 0.02	*R*^2^ = 0.28
Frontal gray matter volume	Left	*R*^2^ = 0.08	*R*^2^ = 0.05	*R*^2^ = 0.02	*R*^2^ = 0.05	*R*^2^ = 0.03	*R*^2^ = 0.44
	Right	*R*^2^ = 0.09	*R*^2^ = 0.07	*R*^2^ = 0.04	*R*^2^ = 0.035	*R*^2^ = 0.001	*R*^2^ = 0.13
Parietal gray matter volume	Left	*R*^2^ = 0.16^∗^	*R*^2^ = 0.09	*R*^2^ = 0.17	*R*^2^ = 0.07	*R*^2^ = 0.06	*R*^2^ = 0.007
	Right	*R*^2^ = 0.08	*R*^2^ = 0.14^∗^	*R*^2^ = 0.01	*R*^2^ = 0.01	*R*^2^ = 0.04	*R*^2^ = 0.18
Occipital gray matter volume	Left	*R*^2^ = 0.15^∗^	*R*^2^ = 0.16^∗^	*R*^2^ = 0.23	*R*^2^ = 0.04	*R*^2^ = 0.11	*R*^2^ = 0.05
	Right	*R*^2^ = 0.05^∗^	*R*^2^ = 0.12^∗^	*R*^2^ = 0.31	*R*^2^ = 0.05	*R*^2^ = 0.09	*R*^2^ = 0.0009
Temporal gray matter volume	Left	*R*^2^ = 0.06	*R*^2^ = 0.02	*R*^2^ = 0.29	*R*^2^ = 0.05	*R*^2^ = 0.01	*R*^2^ = 0.14
	Right	*R*^2^ = 0.02	*R*^2^ = 0.007	*R*^2^ = 0.38	*R*^2^ = 0.001	*R*^2^ = 0.009	*R*^2^ = 0.005
Subcortical gray matter volume	Left	*R*^2^ = 0.004	*R*^2^ = 0.02	*R*^2^ = 0.05	*R*^2^ = 0.005	*R*^2^ = 0.04	*R*^2^ = 0.002
	Right	*R*^2^ = 0.06	*R*^2^ = 0.04	*R*^2^ = 0.12	*R*^2^ = 0.008	*R*^2^ = 0.02	*R*^2^ = 0.06
Frontal white matter volume	Left	*R*^2^ = 0.01	*R*^2^ = 1.9 × 10^−6^	*R*^2^ = 0.17	*R*^2^ = 0.009	*R*^2^ = 0.08	*R*^2^ = 0.07
	Right	*R*^2^ = 0.07	*R*^2^ = 0.006	*R*^2^ = 0.29	*R*^2^ = 0.008	*R*^2^ = 0.05	*R*^2^ = 0.05
Parietal white matter volume	Left	*R*^2^ = 0.02	*R*^2^ = 0.002	*R*^2^ = 0.22	*R*^2^ = 0.009	*R*^2^ = 1.7 × 10^−5^	*R*^2^ = 0.002
	Right	*R*^2^ = 0.04	*R*^2^ = 0.002	*R*^2^ = 0.01	*R*^2^ = 003	*R*^2^ = 0.007	*R*^2^ = 0.05
Occipital white matter volume	Left	*R*^2^ = 0.0009	*R*^2^ = 0.002	*R*^2^ = 0.11	*R*^2^ = 0.09	*R*^2^ = 0.04	*R*^2^ = 0.24
	Right	*R*^2^ = 0.001	*R*^2^ = 0.002	*R*^2^ = 0.42	*R*^2^ = 0.01	*R*^2^ = 0.0007	*R*^2^ = 0.03
Temporal white matter volume	Left	*R*^2^ = 0.005	*R*^2^ = 0.0002	*R*^2^ = 0.002	*R*^2^ = 0.01	*R*^2^ = 0.02	*R*^2^ = 0.01
	Right	*R*^2^ = 2.4 × 10^−9^	*R*^2^ = 0.09	*R*^2^ = 0.05	*R*^2^ = 0.02	*R*^2^ = 0.01	*R*^2^ = 0.03
Subcortical white matter volume	Left	*R*^2^ = 0.2^∗^	*R*^2^ = 0.16	*R*^2^ = 0.06	*R*^2^ = 0.03	*R*^2^ = 0.12	*R*^2^ = 0.02
	Right	*R*^2^ = 0.2^∗^	*R*^2^ = 0.09	*R*^2^ = 0.68	*R*^2^ = 0.03	*R*^2^ = 0.06	*R*^2^ = 0.13


*^∗^p < 0.05. Colors indicate inconsistents results, with statistically significant and non statistically significant differences respectively in green and orange.*

### Variability of the Results and Processing Pipeline Methods

**FIGURE 4:**
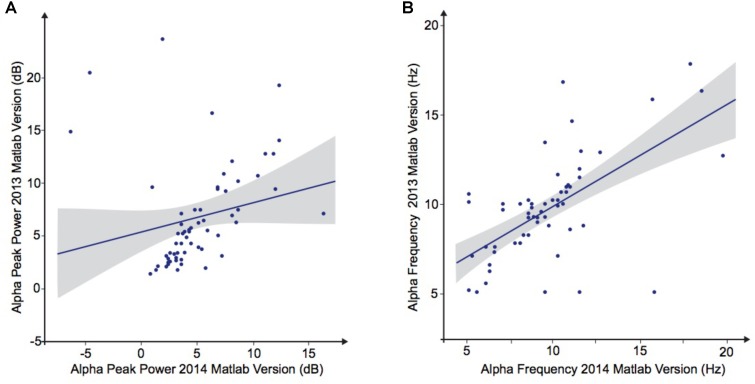
Alpha waves characteristics analyzed by Matlab 2013 versus Matlab 2014. **(A)** Alpha peak power (dB). **(B)** Alpha frequency (Hz).

To explore the dependence of our results to the pipelines of analysis, we re-extracted the alpha frequency and its peak power from our dataset using first Matlab 2013a with the Signal Processing toolbox, and then Python with the MNE Python library. We compared these extractions to those obtained with Matlab 2014 (**Figure 4**). No significant difference of alpha frequency and its peak power was found between groups. Only the pipeline with Matlab 2014 revealed a developmental trajectory for alpha frequency in the TD group. Furthermore, the relationship between left and right occipital gray matter volumes and the alpha waves frequency appeared to be consistent among the two versions of Matlab, independently from the use of the Signal Processing toolbox (**Table 4**). Surprisingly none of those significant associations reported with Matlab was confirmed with Python (**Table 5**).

**Table 5:** Bivariate regression analysis regarding the association of alpha characteristics (frequency and power) related to participants age.

		Matlab 2014	Matlab 2013	Python 2.7
Alpha frequency	ASD	*R*^2^ = 0.001	*R*^2^ = 0.01	*R*^2^ = 0.01
	TD	*R*^2^ = 0.001	*R*^2^ = 0.1^∗^	*R*^2^ = 0.04
Alpha power	ASD	*R*^2^ = 0.08	*R*^2^ = 0.06	*R*^2^ = 0.08
	TD	*R*^2^ = 0.0001	*R*^2^ = 0.08	*R*^2^ = 0.02

*ASD, autism spectrum disorder; TD, typically developing participants. ^∗^p < 0.05.*

## Discussion

A personalized medicine approach in ASD will partially depend on the identification of robust biomarkers that could provide relevant information about its physiopathology or predict the response to treatment. In this way, EEG provides a low-cost but efficient access to brain activity (specifically the postsynaptic potential) with a temporal resolution that go beyond most of the neuroimaging technics (such as functional magnetic resonance imaging) (Buzsáki et al., 2012). Numerous studies pointed toward the alpha waves abnormalities as a robust markers of social dysfunction, especially in ASD (Mathewson et al., 2012; Edgar et al., 2015; Dickinson et al., 2017). Studies reported abnormal frequency or peak power at rest in patients with ASD (Murias et al., 2007) and a deficit in alpha waves suppression in an observed social-communication action condition (Oberman et al., 2005; Dumas et al., 2014b; Hobson and Bishop, 2016). However inconsistency among reports questioned the reliability of these results and need a better scrutiny of the methodological and clinical heterogeneity (Takahashi, 2013). Collecting a larger sample size than most studies of the field, we developed a systematic analysis pipeline aiming to control the detection of the alpha characteristics and potential-related anatomical features that may influence the EEG signal. Nor linear regression neither non-linear normative model did allow to identify a significant relationship between the peak power or the frequency of the alpha waves and the presence of autistic symptoms. Similarly, we observed the alpha characteristics were not significantly influenced by the volume of the white/gray cortical/subcortical brain regions.

Our results thus contrasted with early studies supporting either decreased (Cantor et al., 1986; Dawson et al., 1995; Shephard et al., 2018) or increased (Ogawa et al., 1982; Chan and Leung, 2006; Murias et al., 2007) power in alpha waves activity. Those discrepancies among studies may rely on a higher variability of brain patterns in ASD patients compared to TD participants (Hahamy et al., 2015), with even sometimes the juxtaposition of opposite effects (at the sample level) (Jung et al., 2017). Interestingly, the variability appears correlated to the severity of autistic symptoms entailing greater deviations, both positive and negative, in participant with ASD. These results encouraged the development of new approaches like normative models to move away from the traditional case vs. control approach and tackling heterogeneity (Marquand et al., 2016). This also encourages a better account of EEG complexity such as the spatial and electrophysiologic properties of information transfer between brain regions (Mohammad-Rezazadeh et al., 2016). For example, graph methods were recently used to test both the temporal synchronization and the spatial organization of the resting state signal (Zeng et al., 2017). They observed a deficit in synchronization between regions in ASD, mainly affecting the theta (fronto-occipital pathways) and the alpha bands (inter-hemispheric desynchronization), associated with a more significant deficit in the local clustering than in the long-range connections.

In contrast to our initial hypothesis, we did not observe a specific relationship between the thalamus and the alpha characteristics (Zotev et al., 2018). However, there was a trend for an association between the striatum and the alpha frequency: the left putamen volume located in its dorsal region (*p* = 0.04) and the left nucleus accumbens from its region were both associated with alpha frequency (*p* = 0.002). These results provided additional evidence suggesting the involvement of the striatum in low-frequency oscillations. At rest, a recent study showed the fMRI BOLD activity of the posterior nodes of the default mode network (which includes the dorsal striatum) was significantly correlated to the alpha waves oscillations (Zotev et al., 2018). Also, theta-alpha oscillations correlate with the striatum activity in recollection and reinforcement learning tasks (Herweg et al., 2016), in line with recent EEG results showing a dynamical interplay between reinforcement learning and memory processes (Collins and Frank, 2018). In our study, we also observed that the volumes of the left parietal gray matter (*p* = 0.02) and the bilateral occipital gray matter (respectively, *p* = 0.03 and *p* = 0.05) were associated with the alpha waves frequency. These results corroborated those obtained by magnetoencephalography showing that the alpha activity was associated with part of the occipital lobe (around the Calcarine Sulcus region) and the parietal association cortices (Edgar et al., 2015). Specifically in patients with ASD, these two distinct regions displayed specific pattern of activation in the alpha band, suggesting an intra-parietal or intra-occipital functional dysconnectivity, but also between these two brain structures or between these two regions and widespread brain regions (Ye et al., 2014). The functional dysconnectivity between occipital and parietal regions, which are involved in the observation of human biological motion, may result in an abnormal information processing in ASD (Dumas et al., 2014b; Keehn et al., 2017).

One of the aims of the study was also to further explore the developmental effect on alpha waves, i.e., the effect on age on the alpha frequency and power, suggesting a major interaction between both. The linear method we used first with Matlab 2013 found no statistically significant difference between the ASD and TD groups, nor the effect of age. However, repeated analyses with Matlab 2014 only found a developmental trajectory for the alpha peak frequency (*r*^2^ = 0.1) in the TD group (**Table 4**) which were coherent with developmental differences already reported, namely an increase of the alpha frequency and power with the age of the participant (Dustman et al., 1999; Marshall et al., 2002).

Our sample size did not account for a difference in this trend contrary to previous reports (Tierney et al., 2012). Since there was a significant interactions with age in the neurotypical population, we nevertheless developed a non-parametric normative models aiming at renormalizing alpha characteristics by taking into account potential non-linear effects of age (Marquand et al., 2016). Even after such correction, we did not observe any difference between the two groups.

Phenotypic heterogeneity of ASD might be the cause for the lack of significant association despite our methodological account of the confounding effects of brain volume and age variability. The difference of deviation from normal distribution after non-parametric normative modeling reflects this increased heterogeneity in both alpha frequency and amplitude. Autism, as a spectrum, is known to display strong heterogeneity. For instance, Charman et al. (2017) with the largest longitudinal cohort in autism research in Europe (LEAP) highlighted how this phenotypic heterogeneity include variations both in core ASD symptom severity and in commonly co-occurring psychiatric symptoms, even with a systematic account of sex, age, and IQ. Other studies reported how this heterogeneity is also observable at the genetic (Huguet et al., 2013), neuroanatomical (Lenroot and Giedd, 2006; Lefebvre et al., 2015), and neurofunctional (Hahamy et al., 2015; Kitzbichler et al., 2015) levels. Development of stratification biomarkers and international collaboration to gather large cohort is thus a priority to decipher this heterogeneity (Loth et al., 2016). The *reproducibility crisis* recently mediatized in psychology and neurosciences also shows the importance to increase sample sizes (Smith and Nichols, 2018), the level of statistical practices (Kriegeskorte et al., 2009), and the publication of negative results (Button et al., 2013). Here we also showed that the choice of software also introduced another form of heterogeneity, leading to non-replicable results on the same data. In our case, the proprietary software Matlab and the EEGlab toolbox were introducing an unexpected change in spectral analyses depending on the presence of the Signal Processing toolbox. We addressed this issue in bioinformatics recently (Kim et al., 2018) and neuroinformatics is not an exception to this underestimated problem. While there is not much discussion of the EEG literature, the observations that different operating system for deriving neuroanatomical volume from automated brain segmentation might be a source of heterogeneity have been already described in the MRI literature (Gronenschild et al., 2012; Glatard et al., 2015).

Another source of confound may lie in the measure considered here. Despite alpha being a tentative biomarker because of its link with social cognition (Tognoli et al., 2007; Dumas et al., 2010) and its robust detection and ability to classify other disorders (Engemann and Gramfort, 2015), disentangling ASD from neurotypical population may require the use of more advanced metrics such as functional connectivity (Murias et al., 2007; Khan et al., 2013) or entropy (Bosl et al., 2011; Zeng et al., 2017). Even the structure of alpha rhythm appears more complex than expected with fine-grained sub-components differing in frequency and spatial characteristics (Barzegaran and Knyazeva, 2017). A better understanding of the alpha rhythm – in both health and diseases – will thus also require identification of those components through their differential dynamics and source reconstruction.

## Conclusion

The key findings of our study are the confirmation of the higher variability in ASD group thanks to the normative models’ approach, and the demonstration that variation of pipeline and software could lead to large inconsistency, even on the same data. This may explain our inability to replicate previous results supporting differences in alpha between ASD and controls. This also suggests the need for more sophisticated analytic approaches, although they may require higher-density EEG recording, finer grain anatomical measures, and better experimental design.

Identify novel paradigms may indeed represent the most promising development to uncover relevant biomarkers in ASD. Beside a more precise account of brain activity at both temporal and spatial scales (Dumas et al., 2014b), the challenge will be to study brain activity in individuals with ASD during relevant ecological tasks such as real-time social interaction (Dumas et al., 2014a). We recently proposed to explore the EEG physiological basis of social interactions in specific and restrain conditions, i.e., by studying the interactions between a human and an empirically grounded computational model of human brain and behavior (Dumas et al., 2014b). Participants had to interact directly with a virtual partner, which reacts in real time to the behavior of the participant. Preliminary findings in adults from the general population led to the identification of electrophysiological features that would have been not apparent in real-time human social interactions and allowed the detection of their neuroanatomical brain correlates (Dumas et al., 2014b). Similar explorations in patients with ASD will probably provide new avenues in the field.

## Author Contributions

AL collected all the datas, and participated in the analysis and writing. RD participated in the methods, analysis, writing, and relecture. CD directed the EEG recording and made it possible to collect the EEG datas. FA worked on the inclusion of patients and their clinical exploration. AB used to collect MRI datas. DG and TB participated in the methods and relecture. RT analyzed MRI datas. GD participated in the analysis (in particular normative models) and writing.

## Conflict of Interest Statement

The authors declare that the research was conducted in the absence of any commercial or financial relationships that could be construed as a potential conflict of interest.

## Supplementary Material

FIGURE S1 Inclusion participants pipeline.

FIGURE S2 Preprocessing and processing chain of the data.

FIGURE S3 Agreement charts for comparing control quality by independent raters (Bangdiwala’s test).

FIGURE S4 Linear regression to model the relationship between the peak frequency or the power of Alpha waves and each structural brain volumes.
