# Supplementary figures and images for: Alpha Waves as a Neuromarker of Autism Spectrum Disorder: The Challenge of Reproducibility and Heterogeneity
Source: Front Neurosci. 2018 Oct 1;12:662. doi: 10.3389/fnins.2018.00662 (PMC6174243; doi:10.3389/fnins.2018.00662)

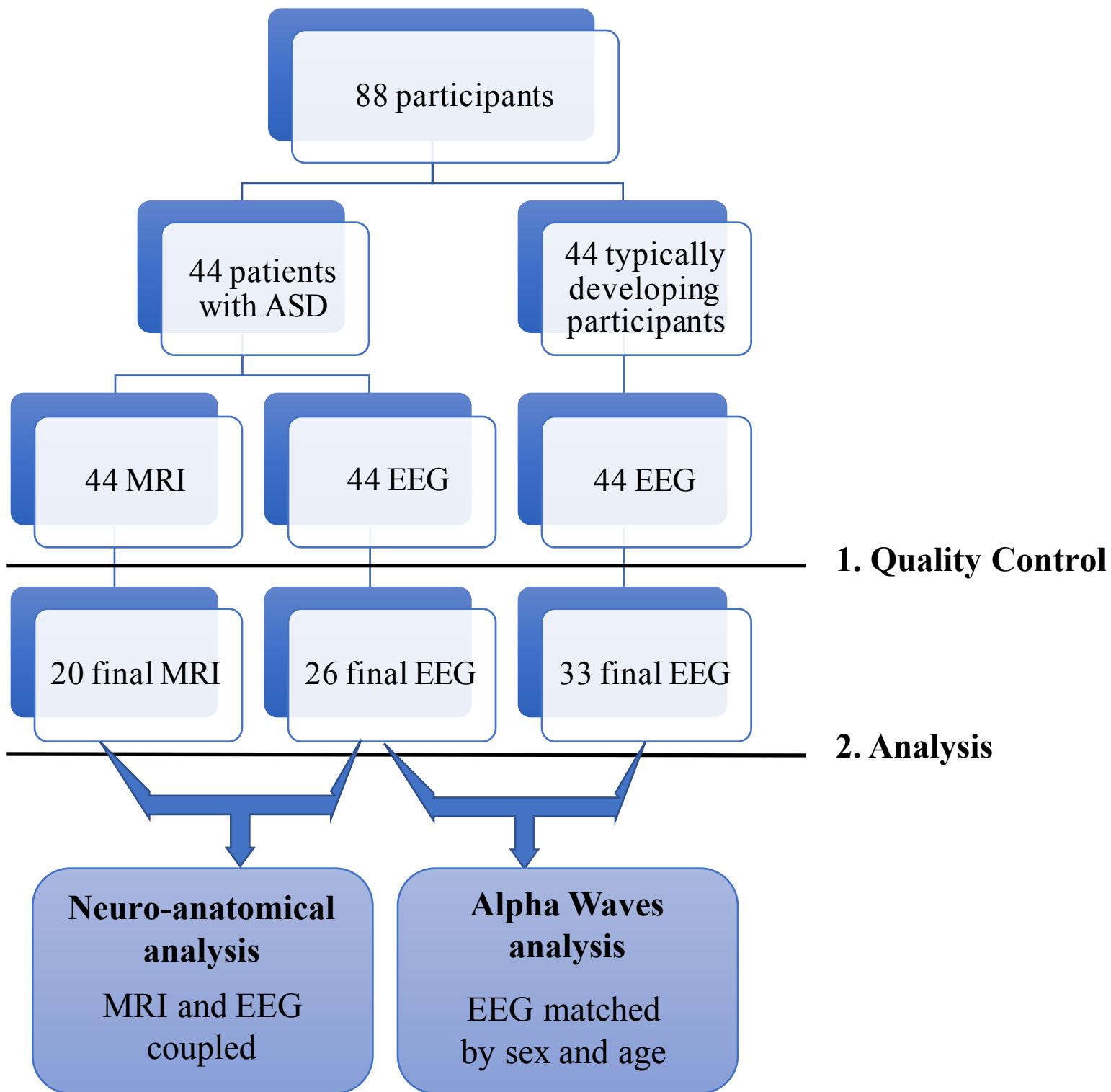

Supplement: FIGURE S1 — Inclusion participants pipeline. [file Data_Sheet_1.PDF]

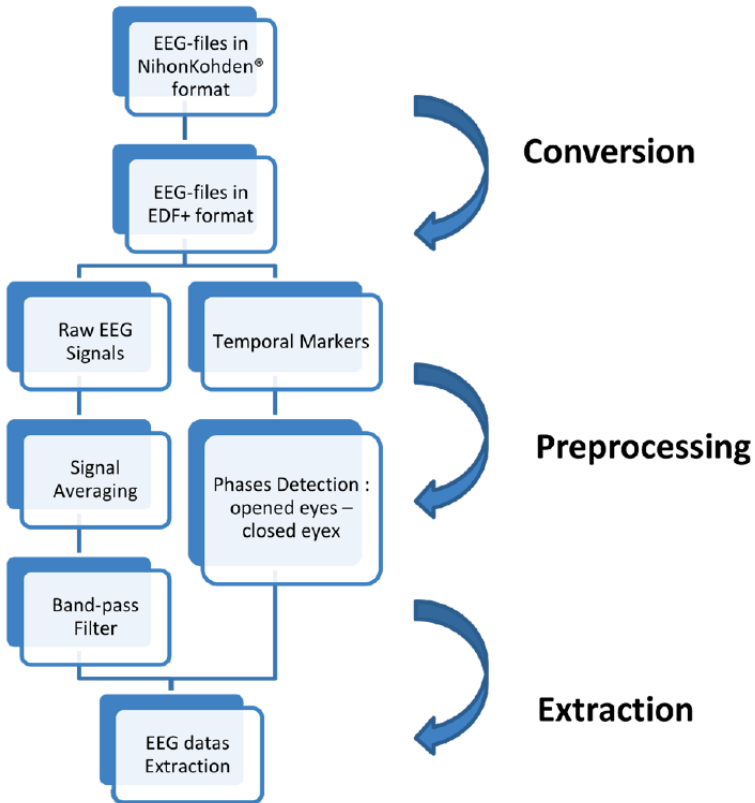

Supplement: FIGURE S2 — Preprocessing and processing chain of the data. [file Data_Sheet_2.PDF]

**Number of EEG**

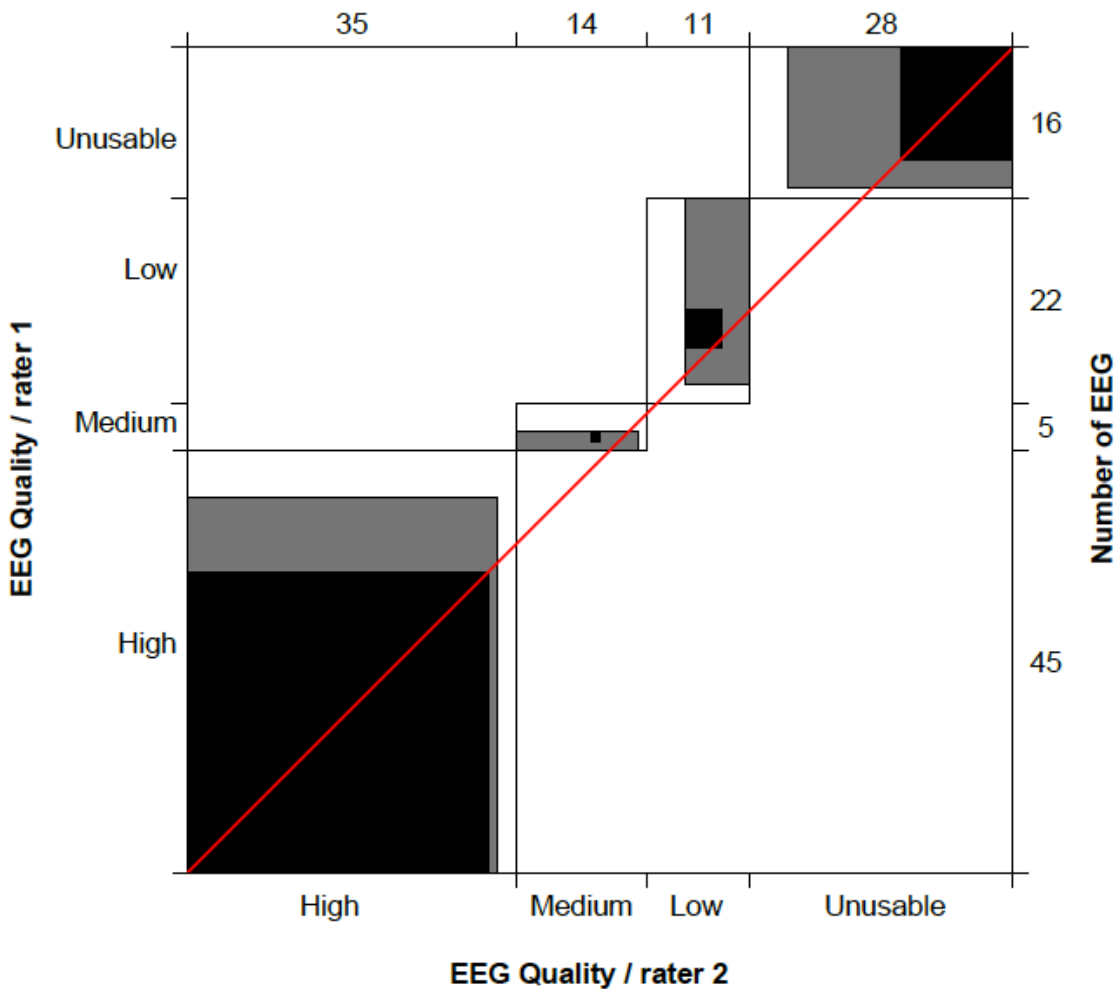

Supplement: FIGURE S3 — Agreement charts for comparing control quality by independent raters (Bangdiwala’s test). [file Data_Sheet_3.PDF]

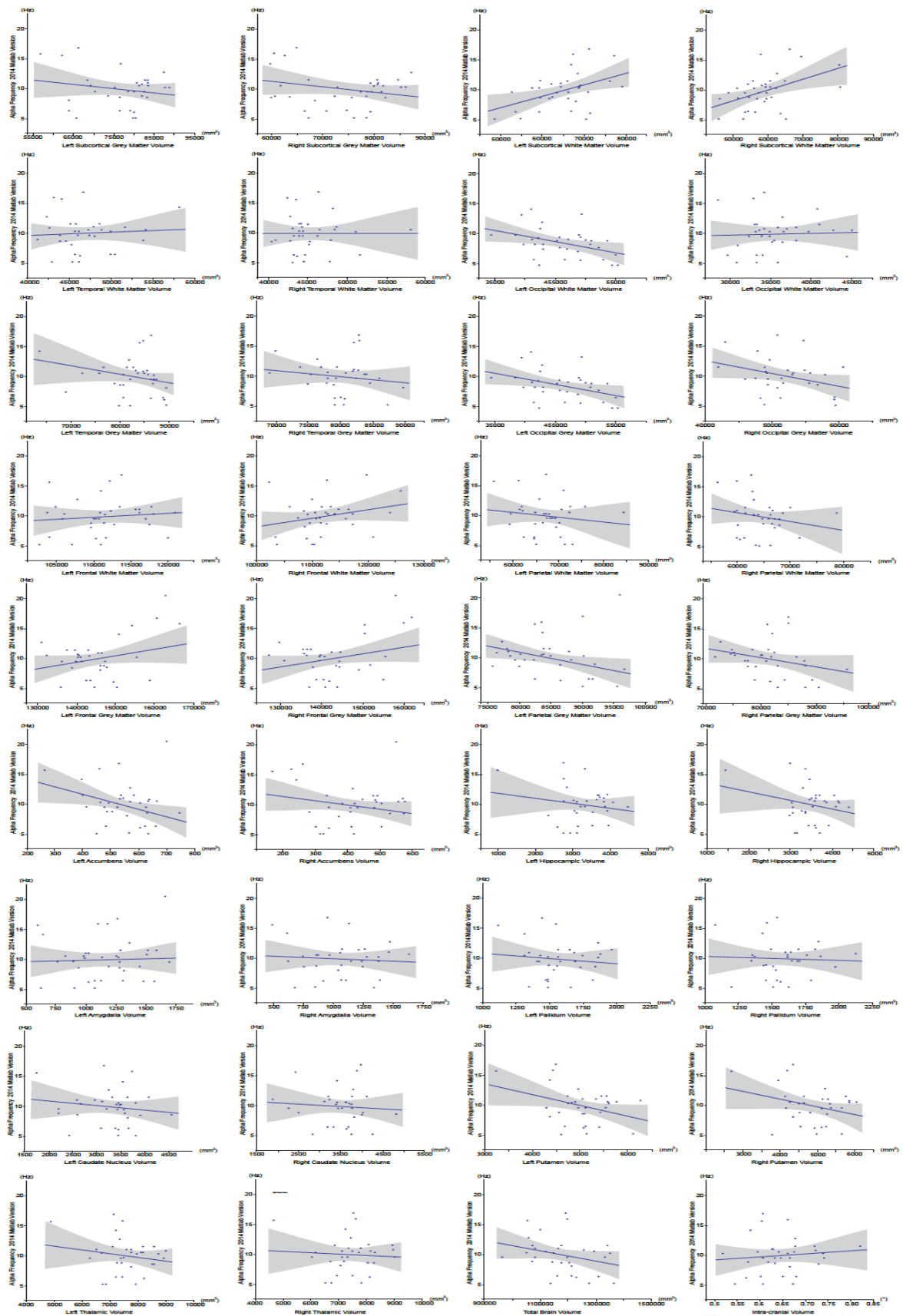

Supplement: FIGURE S4 — Linear regression to model the relationship between the peak frequency or the power of Alpha waves and each structural brain volumes. [file Data_Sheet_4.PDF]
